# Probabilistic Entity-Relationship Diagram: A correlation between functional connectivity and spontaneous brain activity during resting state in major depressive disorder

**DOI:** 10.1371/journal.pone.0178386

**Published:** 2017-06-08

**Authors:** Lu Zhang, Lin Shi, Bin Zhang, Lei Zhao, Yuhao Dong, Jing Liu, Zhouyang Lian, Long Liang, Wenbo Chen, Xiaoning Luo, Shufang Pei, Xiaokai Mo, Wenhui Huang, Fusheng Ouyang, Baoliang Guo, Changhong Liang, Shuixing Zhang

**Affiliations:** 1 Department of Radiology, Guangdong General Hospital/Guangdong Academy of Medical Sciences, Guangzhou, Guangdong, P.R. China; 2 Graduate College, Southern Medical University, Guangzhou, Guangdong, P.R. China; 3 Department of Medicine and Therapeutics, The Chinese University of Hong Kong, Shatin, NT, Hong Kong SAR; 4 Chow Yuk Ho Center of Innovative Technology for Medicine, The Chinese University of Hong Kong, Shatin, NT, Hong Kong SAR; National University of Defense Technology College of Mechatronic Engineering and Automation, CHINA

## Abstract

**Background:**

Alterations of functional connectivity (FC) and spontaneous brain activity (SBA) during the resting state has been observed in subjects with major depressive disorder (MDD). Although there are many studies separately describing on the alterations of FC and SBA in major depressive disorder, their correlation are still have not been performed.

**Methods:**

A literature search based on Pubmed and Embase was conducted until 20 April 2016 to identify studies evaluating the correlation for the alterations between functional connectivity and spontaneous brain activity during resting-state in MDD. Meta-analyses were performed using the Probabilistic Entity-Relationship Diagram (PERD) approach to summarize the relationships among multiple factors in an intuitive manner.

**Results:**

A total of 30 studies (747 individuals with MDD and 757 healthy controls) met the inclusion criteria. In this study, we demonstrated that the functional connectivity and spontaneous brain activity, which was quantitatively measured by the primary analysis methods, was decreased in the parahippocampal gyrus, orbitofrontal cortex (OFC) and postcentral gyrus (PCG), and increased in insula and left dorsal medial prefrontal cortex (DMPFC) in MDD patients. Furthermore, we found that MDD patients presented negative correlation alterations both FC and SBA in the default mode network and the dorsal attention network, but positive correlation alterations both FC and SBA in the insular network, executive control network, the salience network and the other network.

**Conclusions:**

Our results first suggested that there were correlation alterations between functional connectivity and spontaneous brain activity during resting-state in patients with MDD. Besides, we applied a recent meta-analysis approach (PERD) to summarize and integrate the inconsistence of the existing findings regarding the network dysfunction of MDD.

## Introduction

Major depressive disorder (MDD) is a common mental disorder, typically characterized by persistent depressed mood, anxiety and dysphoria, alterations of social behavior and sleep abnormalities [1,2]. Moreover, MDD is one of the most severe brain disorders, and it has been highlighted as the 2nd most disabling condition for its impact on the quality of life and its strong association with suicide rates [3]. Although significant progress has been made in understanding the mechanism of MDD and developing relevant treatments, the exact neurophysiological basis of MDD remains unclear and the rates of recurrence remain high [4].

Researchers have become increasingly interested in the role of abnormal large-scale functional activity in the pathophysiology of MDD [4]. Four large-scale networks have garnered much of the attention: the default mode network (DMN), the dorsal attention network (DAN), the executive control network (ECN), and the salience network (SN). The DMN, which is comprised of the posterior cingulate cortex and adjacent precuneus, the medial prefrontal cortex, the medial, lateral, and inferior parietal cortex, and the medial and inferior temporal cortex, is conceptualized as multiple dissociated networks that subserve self-referential internally directed thought [5–7]. In contrast to the DMN, the DAN shows increased synchronization during goal-directed processes and mainly contains the intraparietal sulcus/superior parietal lobule, frontal eye fields, and extrastriate visual areas [8,9]. The ECN, which including the medial frontal gyrus, superior frontal gyrus and the anterior cingulate cortex, is engaged during executive function tasks that require cognitive control and working memory [10]. The SN, which is comprised of the anterior insula, the dorsal anterior cingulate cortex (dACC), the amygdala, the substantia nigra/ventral tegmental area and the thalamus, segregates internal and external stimuli to guide behavior [11,12].

Recently, a resting state fMRI (rs-fMRI) approach, which assesses functional connectivity by identifying the brain regions where the low frequency blood oxygen level dependent (BOLD) signal exhibits temporal coherence, enables direct quantification of interhemispheric functional interactions [13]. This method has been used to study brain disorders including attention deficit hyperactivity disorder (ADHD), schizophrenia, depression, Alzheimer’s disease (AD), mild cognitive impairment, Parkinson disease (PD), epilepsy and posttraumatic stress disorder [14]. However, the literature of the resting state fMRI studies on MDD points to a lack of consistency in the approaches of data collection, analysis and interpretation of the findings. This fact has led to a number of contradictory findings and the lack of an overall consensus on the interpretation of these changes [15].

In this study, we combined a meta-analysis and a large-sample study to further characterize the effect of MDD on functional activity during resting state, and to clarify the associations among alteration patterns measured by different analysis methods. Furthermore, we benefited from the additional features of a recent graphical meta-analysis software, through which the results of the functional measures of MDD group and the control group can be synthetically compared. Areas of divergence can also be estimated, with respect to the difference of activated brain areas between the two groups under resting state. In particular, we aimed to explore and quantify the regions of overlap and divergence so as to advance our understanding of the influence of depression on brain regions under resting state [16].

## Materials and methods

This study was conducted in accordance with the Preferred Reporting Items for Systematic Reviews and Meta-Analyses (PRISMA) statement [17]. Since this meta-analysis did not involve identifiable patient information, no particular ethical considerations were required.

### Database used and criteria

#### Database and keywords

We performed a comprehensive literature search to identify articles investigating the rs-FC in MDD. The Pubmed and Embase databases were searched until 20 April, 2016 without language restriction, using the keywords “Depress*(-ive/-ion)”, “Major Depression”, “Major Depressive Disorder”, “MDD”, “rs-fMRI”, “resting-state fMRI”, “resting-state functional connectivity”, “rs-FC”, and “spontaneous brain activity”.

#### Study selection

Two investigators independently reviewed the title and abstract of all the literatures generated from computerized searches. The online publications identified from the preliminary selection were then reviewed in full text to assess if the studies met the following inclusion criteria:

1. Participants: (*a*) All healthy controls were cognitively intact, and had no history or clinical evidence of dementia; (*b*) All of them had no history of other major psychiatric illness, loss of consciousness, cardiovascular disease, neurological illness, and lifetime alcohol or drug abuse.
2. Intervention: All of the included subjects underwent resting-state functional magnetic resonance imaging (rs-fMRI).
3. Type of study: original research.

The exclusion criteria including:

1. Group duplication or irrelevant publication;
2. Insufficient data for extraction and analysis.

The final inclusion of studies was based on the agreement of both investigators.

#### Data extraction and quality assessment

Two authors extracted data independently. Disagreements were solved by discussion and consultation with a third author. For accuracy analyses, we extracted the following data for every study: author, year of publication, baseline information about the patients (e.g. sample size, mean age, gender), imaging modality, study design (cross-sectional/longitudinal), correlation coefficient (r), p-value and functional connectivity outcome. For quality assessment of included studies, the Newcastle-Ottawa Scale for case control studies was used (www.ohri.ca/programs/clinical_epidemiology/oxford.asp). The scale for case-control studies consisted of 3 items: (1) Selection, (2) Comparability, and (3) Exposure. After these 3 main items, the scales included 8 subitems. All scores are represented out of 9 possible stars (Table 1). A higher score indicates that the individual study was of higher quality.

**Table 1:** Checklist for quality assessment of case-control studies.

*Case-control study*
**Selection**
1. Was the Case Definition Adequate? (e.g. hospital records) (if yes, one star, no star if definition was inadequate or definition was not described)
2. Was the case collected consecutively and representative? (if yes, one star)
3. Was the source of control group same as case group? (if yes, one star, no star if drawn from a different source or the source was not described)
4. Were controls had no history of this outcome(endpoint)and definited explicitly? (if yes, one star)
**Comparability**
5. Adjustment for confounding factors? (if age- and sex-matched, one star, other important factors controlled, one star)
**Exposure**
6. Ascertainment of Exposure: any reliable document or others? (if yes, one star)
7. Same method of ascertainment for cases and controls? (if yes, one star)
8. Was non-response rate same in both groups? (if yes, one star)

Assessment of included trials: low quality, 1–4 stars; high quality, 5–9 stars.

### Generation of the Probabilistic Entity-Relationship Diagram (PERD) of MDD

The PERD (http://www.med-perd.org/) of MDD was a colored diagram that summarizes the imaging biomarkers of MDD and visualizes their relationships. The nodes denoted the various contributors of MDD, while the edges stood for the corresponding relationships between the nodes. The width and color of the edge were designed to represent the strength of the relationship by integrating reporting times, number of subjects diagnosed with MDD, study design (longitudinal or cross-sectional), correlation coefficient and corresponding significance level (p-value) of the relationship.

#### Width of the edge

The width of edge showed the degree of consensus on a certain correlation. Among the factors describing the relationship of biomarkers, reporting times (T) and the number of MDD subjects (N) indicated the scale of work on the agreement of the relationship, which governed edge width (W). In addition, weighting was used to reflect the study design (cross-sectional or longitudinal) and the size of relationship significance (p-value).

#### Color of the edge

Different colors represented the sign and strength of the correlation. The strength of correlation was illustrated with coded color from yellow to red for positive correlation and green to blue for negative correlation [18].

$$W=W_{0}+\Sigma_{i=1}^{T}Asign\left(r_{i}\right)N_{i}\alpha_{i}\beta_{i}$$

The equation was used to calculate the width of the edge. W_0_ was the default width of edge for observed correlations (W_0_ = 0.5pt). The weight A was experiential designed to adjust the appearance of the edges. The combined term on the right contained the results of various reports on the same relationship, and the parameter is referred to the result of a single report. The details of parameterization were further explained in Table 2.

**Table 2:** The details of parameterization for the relationship of MDD biomarkers.

Factors	Description
Report times(T)	The number of current studies regarding the same relationship.
Number of MDD subjects(N)	The number of subjects clinical presented with MDD (longitudinal study) or related to a high-risk factor for MDD (cross-sectional study)
Correlation coefficient (r)	This coefficient denotes the degree of correlation between two biomarkers. The sign of r shows the correlation is positive (sign(r) = + 1) or negative (sign(r) = − 1).
Cross-sectional penalty(α)	If the study is cross-sectional, its result should bear a penalty, due to its restriction in observing the conversion in from NC (normal control) to real MDD patient. For cross-sectional study, α = 0.5; for longitudinal study, α = 1. The value of 0.5 in this penalty is determined empirically, and an alternative setting makes little difference with current findings in surveyed literatures.
Significance penalty(β)	This penalty is used to integrate the significance of a finding to width, where β = (0.05/p)^1/2^. We use 0.05 as the reference significance level and introduce the square operation to avoid extreme edge appearance.
Scaling coefficient (A)	This coefficient is empirically set to adjust the width of edge for best appearance. (A = 0.01)

## Results

**Fig 1:**
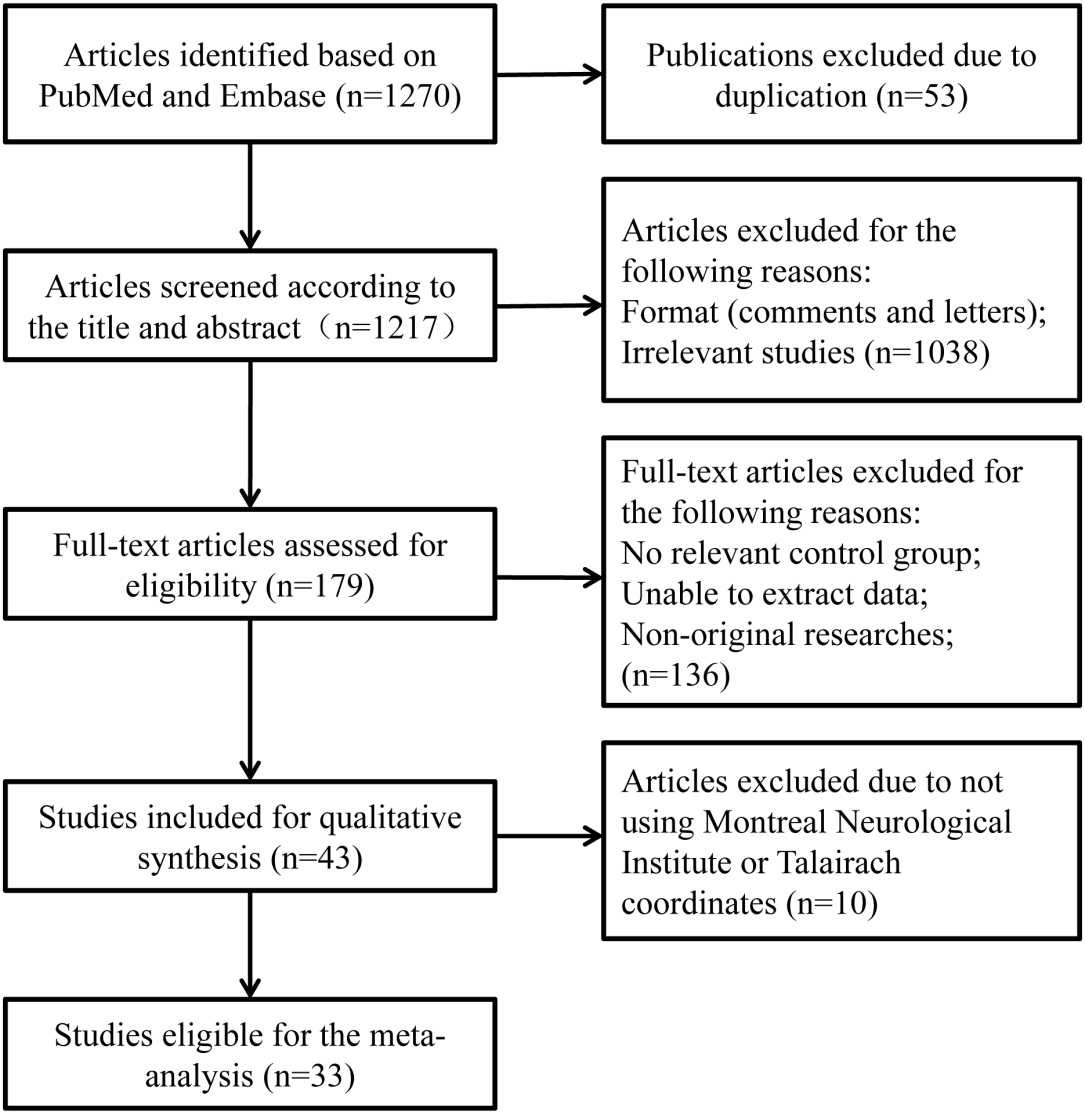
Flow diagram of study selection.

We obtained 1270 publications with the primal literature searching, and 1217 were excluded due to result duplication, irrelevance to the current analysis, non-original research or insufficient data for analysis. Therefore, 43 studies met the initial inclusion criteria consequently. Ten studies were excluded from the final meta-analysis because they did not apply Montreal Neurological Institute or Talairach coordinates. Finally, there were 33 studies eligible for the meta-analysis (Fig 1, Table 3). All of this case-control studies were high quality.

**Table 3:** Summary of the literatures surveyed.

Number	Study ID	Number (CN: pAD)	Mean age (CN: pAD)	Medication	Primary analysis method
(1)	[1]	38:38	32:30	No	Seed-FC
(2)	[2]	22:19	28:24	No	NH(fALFF)
(3)	[19]	41:29	15:16	Partly	Seed-FC (amygdala)
(4)	[20]	24:24	25:24	No	NH
(5)	[21]	17:17	26:24	No	NH(ReHo)
(6)	[22]	49:68	36:35	Partly	NH(ALFF /fALFF)
(7)	[3]	16:16	34:33	No	Seed-FC (insula)
(8)	[23]	11:18	71:64	Partly	Seed-FC(Cerebellar)
(9)	[24]	17:20	19:19	No	NH(fALFF)
(10)	[25]	15:16	15:13	No	NH(ALFF)
(11)	[26]	44:44	28:29	No	NH(fALFF)
(12)	[27]	16:16	34:33	No	NH(ReHo)
(13)	[28]	15:15	29:30	Partly	NH(ReHo)
(14)	[29]	21:18	9:9	No	Seed-FC(whole-brain)
(15)	[30]	42:30	29:26	No	Seed-FC (hippocampus)
(16)	[31]	24:24	25:24	No	NH(fALFF)
(17)	[32]	37:37	20:20	No	Seed-FC
(18)	[33]	32:35	20:20	No	Seed-FC
(19)	[34]	37:14	9:8	No	Seed-FC (amgydala)
(20)	[11]	23:20	33:31	Partly	Seed-FC (whole-brain)
(21)	[35]	21:21	37:38	No	Seed-FC (whole-brain)
(22)	[36]	18:17	35:30	No	Seed-FC
(23)	[37]	44:27	36:38	No	NH(fALFF)
(24)	[38]	7:9	70:68	No	Seed-FC
(25)	[39]	14:14	32:34	No	NH(ReHo)
(26)	[40]	16:14	34:30	No	Seed-FC (whole-brain)
(27)	[41]	24:24	25:24	No	Seed-FC
(28)	[42]	20:20	38:33	No	Seed-FC
(29)	[43]	15:15	67:64	No	NH(ReHo)
(30)	[44]	19:18	66:66	Partly	NH(ReHo)

Note: HC, healthy controls; ReHo, regional homogeneity; (f) ALFF, (fractional) amplitude of low frequency fluctuations; VMHC, voxel-mirrored homotopic connectivity; ICA, independent component analysis; NH, network homogeneity. All studies are cross-sectional and only the data with p < 0.05 was selected.

**Fig 2:**
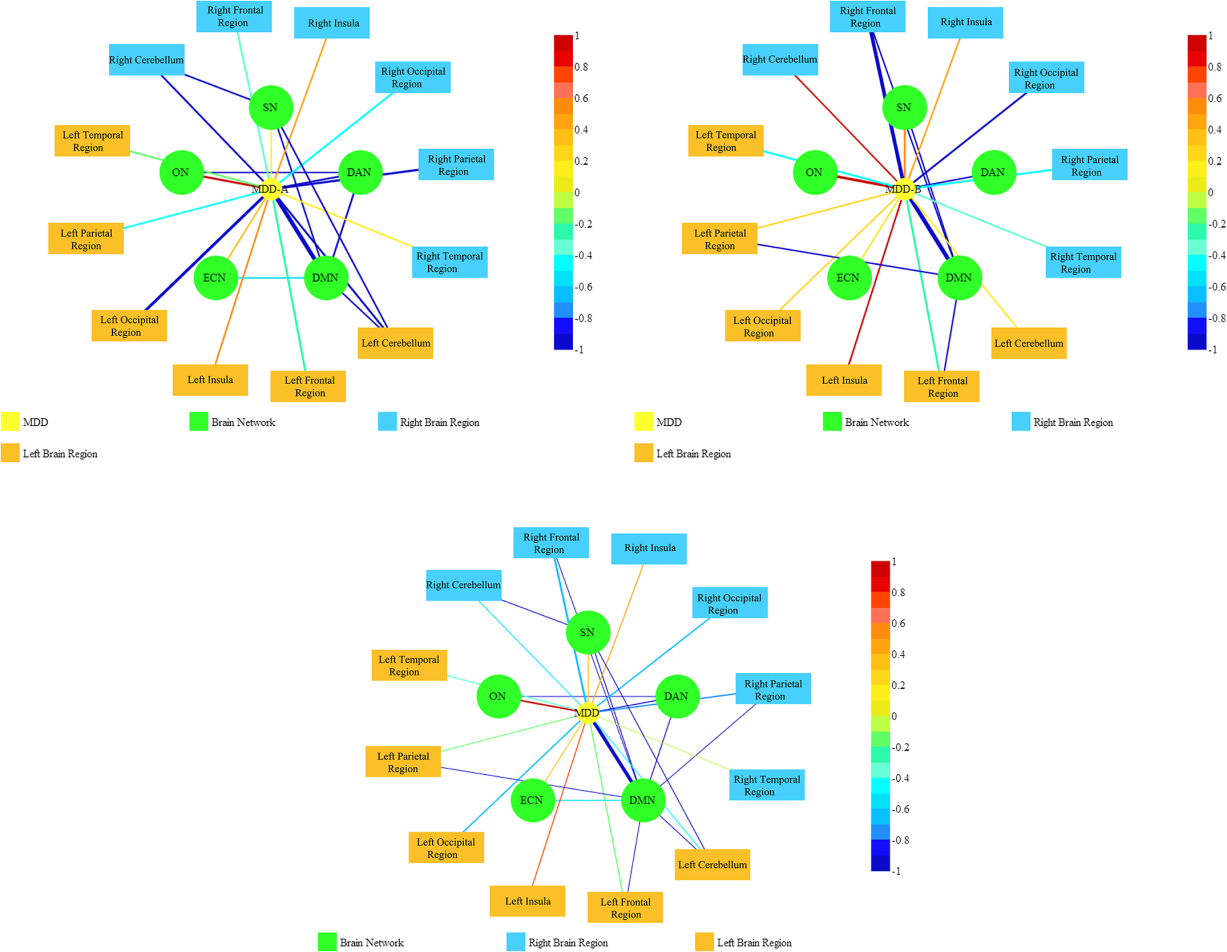
The Probabilistic Entity-Relationship Diagram of MDD. Each point refers to the rs-FC of the brain and the color of each point refers to different categories. Edge widths refer to the degree of consensus on a certain correlation and the color of edge widths refer to the sign and strength of the correlation. (a) The PERD of MDD-A represent rest-stating functional connectivity. (b) The PERD of MDD-B represent spontaneous brain activity. (c) The PERD of MDD represent a combination of them.

The brain functional activity was analyzed by the amplitude of low frequency fluctuations (ALFF), fractional ALFF (fALFF), seed-based functional connectivity, and regional homogeneity (ReHo) of the blood-oxygen- level-dependent (BOLD) signals derived from resting-state functional magnetic resonance imaging (rs-fMRI) [45]. We found that the measures derived from the different analysis methods in MDD patients were all decreased in the parahippocampal gyrus, orbitofrontal cortex (OFC) and postcentral gyrus (PCG), and increased in insula and left dorsal medial prefrontal cortex (DMPFC), compared to the control group. Fig 2a–2c (S1–S3 Appendices) showed the relationship among possible contributing factors of MDD based on the literature findings (Tables 4 and 5). In this diagram, the brain was divided into two categories. The widely used network including the default mode network (DMN), the dorsal attention network (DAN), the executive control network (ECN), the salience network (SN), and the other network (ON), which indicate the areas that were not covered by the former four brain functional networks. The brain regions include frontal region, parietal region, occipital region, temporal region, insula and cerebellum.

**Table 4:** Detailed intra-network of studies included in the meta-analysis.

	NH (ALFF/fALFF/ReHo)		Seed-FC	
Regions	Increase	Decrease	Increase	Decrease
cere.P. lobe		(2,5,9,12,13,29)		
cere.A. lobe	(25)			
Parahip		(2,5,9, 16)		(3)
Hip			(26, 27)	(3)
Brainstem				(3)
OFC		(18,30)		(3)
Precuneus		(9,13,29)	(3, 20)	
L. dmPFC	(4,16, 25)		(8, 22)	
R. dmPFC			(22)	(8, 28)
ITG	(10)	(4,9,24,29)		(27)
R.fusiform	(9, 10)	(4)		
L. fusiform	(18)			(8)
PCG		(5, 13, 29, 30)		(23)
AI	(6)		(20)	
Lingual	(9)	(6)		
DLPFC		(9,29,30)	(20)	(8, 27)
L.IFG		(9,13)		
L.IPG		(13)		(27)
MC		(9,29)		
PCC	(10)	(9)		(26, 28)
ACC			(20)	
R. SMG		(9)		
R.STG	(10)			(28)
L.STG		(13, 29)		(28)
L.SOG	(2)			
Insula	(10,18,30)		(13)	
PG	(10)	(25)		
FG		(2, 23, 25)		
OG		(10,11,12,25)		
TG	(18)	(12)	(23)	(3)
MFG		(10,11,13,30)		
MPFC		(29)		(26)
L.tha		(12)		
AMC	(17)			
PMC		(17)		
Amygdala		(13, 29)		(19)
Ven.stri				(26)

Abbreviations: L/R: left;/right; I/S: inferior/superior; M: medial; A/P: anterior/posterior; DL: dorsolateral; dm: dorsal medial; OG: occipital gyrus; PG: parietal gyrus; FG: frontal gyrus; TG: temporal gyrus; PFC: prefrontal cortex; hip: hippocampal; tha: thalamus; cereb. tonsil: cerebellar tonsil; cere: cerebellum; PCG: postcentral gyrus; MC: motor cortex; SMG: supramarginal gyrus; BA10p: included the ventromedial PFC, dorsal ACC, superior frontal gyrus, PCC, precuneus and angular gyrus, subcortical regions, the thalamus, caudate, and putamen; BA25: subcallosal cortex, frontal pole; Ven.stri: ventral striatum

**Table 5:** Detailed between-nework of studies included in the meta-analysis.

Regions	(1)	(7)	(14)	(15)	(21)	(24)
PCC -dmPFC	−					
PCC-R.IPG	−					
L.tha-cereb.tonsil	−					
Insula- dmPFC		−				
Insula- pulvinar		−				
PCC-MTG			−			
PCC-IPG			−			
PCC-cere			−			
PCC-ACC			+			
R.hip-R.ITG				−		
R.hip- cere				−		
BA10p-R. Iinsula					−	
BA25-ACC					−	
rAI-dACC						−
rAI-SN						−
rAI-R.dlPFC						+
rAI-R.PCC						+

DMN, which was reported with the most detailed anatomical information, was independently visualized with regional findings (Fig 2a–2c). In these diagrams, DMN had significant negative correlation with MDD in terms of edge color, and DMN also presented strong prediction effect on MDD regarding the edge width. Besides, MDD was negative correlation with DAN, but positive correlation with SN, ECN and ON either in functional connectivity or spontaneous brain activity. Furthermore, the presence of MDD was characterized by negative correlation with frontal regions and positive correlation with insula. There were significant discrimination between functional connectivity and spontaneous brain activity in the cerebellum, the occipital, parietal and temporal regions.

MDD was not only associated with intra-network dysfunction, but also contributed to the disturbed interplay between the networks [46]. Within the patients with MDD, negative correlation was found in Fig 2a and 2b between the DMN and the SN. However, negative correlation was just shown in Fig 2a between the DMN and the DAN, ECN, cerebellum, between the SN and the cerebellum, as well as a negative correlation in Fig 2b between the DMN and the frontal regions. The widths and colors of the edges do differ obviously, probably because the studies on these biomarkers are still lacking and that the number of the participants with MDD that involve corresponding biomarkers is very small [4,18].

## Discussion

The presented meta-analyses on resting-state fMRI studies of major depressive disorder (MDD) revealed the strong correlations for alterations of functional connectivity and spontaneous brain activity in key brain regions [47]. To our knowledge, this is the first study that integrates and visualizes the associations between the alteration patterns of functional connectivity and spontaneous brain activity, where the results from different MDD studies were not consistent. With the help of PERD, a recent graphic meta-analysis tool, we found that functional connectivity measured by different analysis methods was decreased in the parahippocampal gyrus, orbitofrontal cortex (OFC) and postcentral gyrus (PCG), and increased in insula and left dorsal medial prefrontal cortex (DMPFC).

The negative correlation of the parahippocampal gyrus in MDD patients was observed in most of the included studies, which was consistent with the deficits in low level sense of well-being and the high level of general depression, dysphoria, and lassitude in MDD. The functional abnormality of the parahippocampal gyrus was also related to emotional symptoms and memory deficit, which partly contribute to emotional dysregulation in patients with MDD [2,19,21,24,31]. The decreased function in the OFC is also in line with the structural and functional deficits within these regions in patients with MDD.

We observed increased functional alteration in the left DMPFC in MDD, which was consistent with abnormal BOLD signals and baseline metabolism or perfusion in this area. The functional abnormality of the parahippocampal gyrus was also related to high level of self-awareness and low level of focus on environmental phenomena, including ongoing events, persons and objects. Moreover, The DMPFC might contribute to the therapeutic mechanism of MDD, where escitalopram treatment could reverse the abnormally increased brain activity in the left DMPFC of MDD patients. Thus, the DMPFC may be another target that mediates the effects of escitalopram treatment on the fronto-limbic circuit [20,23,39].

An analysis on resting-state functional connectivity and spontaneous brain activity in MDD showed negative correlations between MDD and DMN, and between MDD and DAN. Concurrence of positive alterations in major depressive disorder were found in the SN, ECN and ON. The DMN is active and synchronized when the brain is ‘at rest’, while the DAN shows increased synchronization during goal-directed processes. Maybe it’s because the studies on these biomarkers are still lacking and that the number of participants with MDD that involve corresponding biomarkers is very small [11,18,48]. Besides, it is difficult to repeat the results that were obtained from various task-states because of the complicated designs of these studies [28].

The ECN has demonstrated increased resting-state activity, in contrast with the decreased task-based activity in the majority of earlier studies. The divergence in the ECN between increased resting-state activity and decreased task-based activity might be explained by the presence of higher and more volatile activity in these regions at rest, which in turn leads to smaller increases during tasks. However, either increased or decreased functional connectivity has been reported in the ECN in MDD. Although our results did not show detailed changes in the ECN in MDD, the meta-analysis showed increased resting-state activity. The strength of anticorrelation between ECN and DMN was associated with more consistent performance on demanding tasks [36,45,49]. The SN also showed increased resting-state activity, which played a key role in switching between the DMN and executive network to mediate selection of physiologically relevant external and interoceptive signals [46,49,50]. According to previous studies [10,11], the overlaps among networks may be responsible for the same brain functional activity, and could lead to an unavoidable bias.

Inconsistent alterations in different analysis measures have been reported in many regions in MDD patients, either with decreased or increased activity. Our study has demonstrated that there are differences in cerebellum, the occipital regions, parietal regions and temporal regions. We assumed that the differences may be related to structural abnormalities [4,7,51], but these should be supported by more reliable researches. There are also some limitations of this study. First, some the included studies recruited subjects who were on psychotropic medication due to ethical considerations. Thus, we cannot rule out the potential impact of the medication. Second, although the patients from the included studies were asked some questions to confirm the cooperation after the scan, the researchers could not ensure the “resting” state, especially for the patients who engaged in rumination. Third, the literatures involved in this study were cross-sectional without follow up for long-term brain activity at rest [22,45].

## Conclusions

We performed a meta-analysis and a large-sample study to clarify two questions as follows. First, we demonstrated that the functional connectivity measured by primary analysis methods in MDD patients was decreased parahippocampal gyrus, orbitofrontal cortex (OFC) and postcentral gyrus (PCG), and increased in insula and left dorsal medial prefrontal cortex (DMPFC). Second, our findings suggest that MDD patients have negative correlation alterations both functional connectivity and spontaneous brain activity in the default mode network and the dorsal attention network, and positive correlation alterations in the executive control network, the salience network and the other network. These findings may help to expend our knowledge of the pathophysiological of MDD and guide future researches in the related field.

## Supporting information

S1 Appendix The primary data of figure MDD. (PDF)

S2 Appendix The primary data of figure MDD-A. (PDF)

S3 Appendix The primary data of figure MDD-B. (PDF)

S1 Checklist PRISMA checklist. (DOC)

## References

[1] Chen Y, Wang C, Zhu X, Tan Y, Zhong Y (2015) Aberrant connectivity within the default mode network in first-episode, treatment-naive major depressive disorder. J Affect Disord 183: 49–56. 10.1016/j.jad.2015.04.052 26001663

[2] Liu F, Guo W, Liu L, Long Z, Ma C, et al (2013) Abnormal amplitude low-frequency oscillations in medication-naive, first-episode patients with major depressive disorder: a resting-state fMRI study. J Affect Disord 146: 401–406. 10.1016/j.jad.2012.10.001 23116810

[3] Iwabuchi SJ, Peng D, Fang Y, Jiang K, Liddle EB, et al (2014) Alterations in effective connectivity anchored on the insula in major depressive disorder. Eur Neuropsychopharmacol 24: 1784–1792. 10.1016/j.euroneuro.2014.08.005 25219936

[4] Kaiser RH, Andrews-Hanna JR, Wager TD, Pizzagalli DA (2015) Large-Scale Network Dysfunction in Major Depressive Disorder: A Meta-analysis of Resting-State Functional Connectivity. JAMA Psychiatry 72: 603–611. 10.1001/jamapsychiatry.2015.0071 25785575 PMC4456260

[5] Buckner RL, Andrews-Hanna JR, Schacter DL (2008) The brain's default network: anatomy, function, and relevance to disease. Ann N Y Acad Sci 1124: 1–38. 10.1196/annals.1440.011 18400922

[6] Raichle ME, Snyder AZ (2007) A default mode of brain function: a brief history of an evolving idea. Neuroimage 37: 1083–1090; discussion 1097–1089. 10.1016/j.neuroimage.2007.02.041 17719799

[7] Zeng LL, Shen H, Liu L, Wang L, Li B, et al (2012) Identifying major depression using whole-brain functional connectivity: a multivariate pattern analysis. Brain 135: 1498–1507. 10.1093/brain/aws059 22418737

[8] Kim H (2010) Dissociating the roles of the default-mode, dorsal, and ventral networks in episodic memory retrieval. Neuroimage 50: 1648–1657. 10.1016/j.neuroimage.2010.01.051 20097295

[9] Fox MD, Corbetta M, Snyder AZ, Vincent JL, Raichle ME (2006) Spontaneous neuronal activity distinguishes human dorsal and ventral attention systems. Proc Natl Acad Sci U S A 103: 10046–10051. 10.1073/pnas.0604187103 16788060 PMC1480402

[10] Seeley WW, Menon V, Schatzberg AF, Keller J, Glover GH, et al (2007) Dissociable intrinsic connectivity networks for salience processing and executive control. J Neurosci 27: 2349–2356. 10.1523/JNEUROSCI.5587-06.2007 17329432 PMC2680293

[11] Crowther A, Smoski MJ, Minkel J, Moore T, Gibbs D, et al (2015) Resting-state connectivity predictors of response to psychotherapy in major depressive disorder. Neuropsychopharmacology 40: 1659–1673. 10.1038/npp.2015.12 25578796 PMC4915248

[12] Uddin LQ (2015) Salience processing and insular cortical function and dysfunction. Nat Rev Neurosci 16: 55–61. 10.1038/nrn3857 25406711

[13] Wei XH, Ren JL, Liu WH, Yang RM, Xu XD, et al (2015) Increased interhemispheric functional connectivity in college students with non-clinical depressive symptoms in resting state. Neurosci Lett 589: 67–72. 10.1016/j.neulet.2015.01.034 25596443

[14] Song XW, Dong ZY, Long XY, Li SF, Zuo XN, et al (2011) REST: a toolkit for resting-state functional magnetic resonance imaging data processing. PLoS One 6: e25031 10.1371/journal.pone.0025031 21949842 PMC3176805

[15] Wang L, Hermens DF, Hickie IB, Lagopoulos J (2012) A systematic review of resting-state functional-MRI studies in major depression. J Affect Disord 142: 6–12. 10.1016/j.jad.2012.04.013 22858266

[16] Palmer SM, Crewther SG, Carey LM (2014) A meta-analysis of changes in brain activity in clinical depression. Front Hum Neurosci 8: 1045 10.3389/fnhum.2014.01045 25642179 PMC4294131

[17] Moher D, Liberati A, Tetzlaff J, Altman DG (2010) Preferred reporting items for systematic reviews and meta-analyses: the PRISMA statement. Int J Surg 8: 336–341. 10.1016/j.ijsu.2010.02.007 20171303

[18] Shi L, Zhao L, Wong A, Wang D, Mok V (2015) Mapping the Relationship of Contributing Factors for Preclinical Alzheimer's Disease. Sci Rep 5: 11259 10.1038/srep11259 26190794 PMC4507140

[19] Kathryn R. Cullen M, Melinda K. Westlund B, Bonnie Klimes-Dougan P (2014) <Abnormal amygdala resting-state functional connectivity in adolescent depression..pdf>. 10.1001/jamapsychiatry.2014.1087 PMC4378862 25133665

[20] Guo W, Liu F, Zhang J, Zhang Z, Yu L, et al (2014) Abnormal default-mode network homogeneity in first-episode, drug-naive major depressive disorder. PLoS One 9: e91102 10.1371/journal.pone.0091102 24609111 PMC3946684

[21] Guo WB, Liu F, Xue ZM, Yu Y, Ma CQ, et al (2011) Abnormal neural activities in first-episode, treatment-naive, short-illness-duration, and treatment-response patients with major depressive disorder: a resting-state fMRI study. J Affect Disord 135: 326–331. 10.1016/j.jad.2011.06.048 21782246

[22] Liu CH, Ma X, Song LP, Fan J, Wang WD, et al (2015) Abnormal spontaneous neural activity in the anterior insular and anterior cingulate cortices in anxious depression. Behav Brain Res 281: 339–347. 10.1016/j.bbr.2014.11.047 25513974

[23] Alalade E, Denny K, Potter G, Steffens D, Wang L (2011) Altered cerebellar-cerebral functional connectivity in geriatric depression. PLoS One 6: e20035 10.1371/journal.pone.0020035 21637831 PMC3102667

[24] Wei X, Shen H, Ren J, Li X, Xu X, et al (2014) Altered resting-state connectivity in college students with nonclinical depressive symptoms. PLoS One 9: e114603 10.1371/journal.pone.0114603 25502215 PMC4264752

[25] Gong Y, Hao L, Zhang X, Zhou Y, Li J, et al (2014) Case-control resting-state fMRI study of brain functioning among adolescents with first-episode major depressive disorder. Shanghai Arch Psychiatry 26: 207–215. 25317007 10.3969/j.issn.1002-0829.2014.04.004 PMC4194003

[26] Guo W, Liu F, Yu M, Zhang J, Zhang Z, et al (2015) Decreased regional activity and network homogeneity of the fronto-limbic network at rest in drug-naive major depressive disorder. Aust N Z J Psychiatry 49: 550–556. 10.1177/0004867415577978 25788499

[27] Peng DH, Jiang KD, Fang YR, Xu YF, Shen T, et al (2011) Decreased regional homogeneity in major depression as revealed by resting-state functional magnetic resonance imaging. Chin Med J (Engl) 124: 369–373. 21362335

[28] Liu Z, Xu C, Xu Y, Wang Y, Zhao B, et al (2010) Decreased regional homogeneity in insula and cerebellum: a resting-state fMRI study in patients with major depression and subjects at high risk for major depression. Psychiatry Res 182: 211–215. 10.1016/j.pscychresns.2010.03.004 20493670

[29] Gaffrey MS, Luby JL, Botteron K, Repovs G, Barch DM (2012) Default mode network connectivity in children with a history of preschool onset depression. J Child Psychol Psychiatry 53: 964–972. 10.1111/j.1469-7610.2012.02552.x 22519864 PMC3437619

[30] Cao X, Liu Z, Xu C, Li J, Gao Q, et al (2012) Disrupted resting-state functional connectivity of the hippocampus in medication-naive patients with major depressive disorder. J Affect Disord 141: 194–203. 10.1016/j.jad.2012.03.002 22460056

[31] Guo W, Liu F, Zhang J, Zhang Z, Yu L, et al (2013) Dissociation of regional activity in the default mode network in first-episode, drug-naive major depressive disorder at rest. J Affect Disord 151: 1097–1101. 10.1016/j.jad.2013.09.003 24074717

[32] Zhu X, Wang X, Xiao J, Liao J, Zhong M, et al (2012) Evidence of a dissociation pattern in resting-state default mode network connectivity in first-episode, treatment-naive major depression patients. Biol Psychiatry 71: 611–617. 10.1016/j.biopsych.2011.10.035 22177602

[33] Zhang X, Zhu X, Wang X, Zhu X, Zhong M, et al (2014) First-episode medication-naive major depressive disorder is associated with altered resting brain function in the affective network. PLoS One 9: e85241 10.1371/journal.pone.0085241 24416367 PMC3887023

[34] Luking KR, Repovs G, Belden AC, Gaffrey MS, Botteron KN, et al (2011) Functional connectivity of the amygdala in early-childhood-onset depression. J Am Acad Child Adolesc Psychiatry 50: 1027–1041.e1023. 10.1016/j.jaac.2011.07.019 21961777 PMC3185293

[35] Sawaya H, Johnson K, Schmidt M, Arana A, Chahine G, et al (2015) Resting-state functional connectivity of antero-medial prefrontal cortex sub-regions in major depression and relationship to emotional intelligence. Int J Neuropsychopharmacol 18. 10.1093/ijnp/pyu112 PMC4438550 25744282

[36] Sheline YI, Price JL, Yan Z, Mintun MA (2010) Resting-state functional MRI in depression unmasks increased connectivity between networks via the dorsal nexus. Proc Natl Acad Sci U S A 107: 11020–11025. 10.1073/pnas.1000446107 20534464 PMC2890754

[37] Lai CH, Wu YT (2015) The patterns of fractional amplitude of low-frequency fluctuations in depression patients: the dissociation between temporal regions and fronto-parietal regions. J Affect Disord 175: 441–445. 10.1016/j.jad.2015.01.054 25679198

[38] Genevieve S. Yuen FMG-D, Matthew J. Hoptman2 (2014) <The salience network in the apathy of late-life depression..pdf>. 10.1002/gps.4171 PMC4197060 24990625

[39] Wang L, Li K, Zhang Q, Zeng Y, Dai W, et al (2014) Short-term effects of escitalopram on regional brain function in first-episode drug-naive patients with major depressive disorder assessed by resting-state functional magnetic resonance imaging. Psychol Med 44: 1417–1426. 10.1017/S0033291713002031 23942213

[40] Hamilton JP, Chen G, Thomason ME, Schwartz ME, Gotlib IH (2011) Investigating neural primacy in Major Depressive Disorder: multivariate Granger causality analysis of resting-state fMRI time-series data. Mol Psychiatry 16: 763–772. 10.1038/mp.2010.46 20479758 PMC2925061

[41] Guo W, Liu F, Liu J, Yu L, Zhang Z, et al (2013) Is there a cerebellar compensatory effort in first-episode, treatment-naive major depressive disorder at rest? Prog Neuropsychopharmacol Biol Psychiatry 46: 13–18. 10.1016/j.pnpbp.2013.06.009 23800464

[42] van Tol MJ, Li M, Metzger CD, Hailla N, Horn DI, et al (2014) Local cortical thinning links to resting-state disconnectivity in major depressive disorder. Psychol Med 44: 2053–2065. 10.1017/S0033291713002742 24176247

[43] Liu F, Hu M, Wang S, Guo W, Zhao J, et al (2012) Abnormal regional spontaneous neural activity in first-episode, treatment-naive patients with late-life depression: a resting-state fMRI study. Prog Neuropsychopharmacol Biol Psychiatry 39: 326–331. 10.1016/j.pnpbp.2012.07.004 22796277

[44] Ma Z, Li R, Yu J, He Y, Li J (2013) Alterations in regional homogeneity of spontaneous brain activity in late-life subthreshold depression. PLoS One 8: e53148 10.1371/journal.pone.0053148 23301035 PMC3534624

[45] Xu Y, Zhuo C, Qin W, Zhu J, Yu C (2015) Altered Spontaneous Brain Activity in Schizophrenia: A Meta-Analysis and a Large-Sample Study. Biomed Res Int 2015: 204628 10.1155/2015/204628 26180786 PMC4477065

[46] Wei M, Qin J, Yan R, Bi K, Liu C, et al (2015) Association of resting-state network dysfunction with their dynamics of inter-network interactions in depression. J Affect Disord 174: 527–534. 10.1016/j.jad.2014.12.020 25556670

[47] Hannawi Y, Lindquist MA, Caffo BS, Sair HI, Stevens RD (2015) Resting brain activity in disorders of consciousness: a systematic review and meta-analysis. Neurology 84: 1272–1280. 10.1212/WNL.0000000000001404 25713001 PMC4366089

[48] Raichle ME (2011) The restless brain. Brain Connect 1: 3–12. 10.1089/brain.2011.0019 22432951 PMC3621343

[49] Moran LV, Tagamets MA, Sampath H, O'Donnell A, Stein EA, et al (2013) Disruption of anterior insula modulation of large-scale brain networks in schizophrenia. Biol Psychiatry 74: 467–474. 10.1016/j.biopsych.2013.02.029 23623456 PMC3735654

[50] Belleau EL, Taubitz LE, Larson CL (2015) Imbalance of default mode and regulatory networks during externally focused processing in depression. Soc Cogn Affect Neurosci 10: 744–751. 10.1093/scan/nsu117 25274576 PMC4420753

[51] Ma Q, Zeng LL, Shen H, Liu L, Hu D (2013) Altered cerebellar-cerebral resting-state functional connectivity reliably identifies major depressive disorder. Brain Res 1495: 86–94. 10.1016/j.brainres.2012.12.002 23228724

