# Supplementary material for: Probabilistic Entity-Relationship Diagram: A correlation between functional connectivity and spontaneous brain activity during resting state in major depressive disorder
Source: PLoS One. 2017 Jun 8;12(6):e0178386. doi: 10.1371/journal.pone.0178386 (PMC5464553; doi:10.1371/journal.pone.0178386)
Supplement: S1 Appendix — (PDF) [file pone.0178386.s001.pdf]

major facto	related factor	cross/longi	imaging m	No of Nor	corrlation v	p value
DMN	MDD	1	rs-fMRI	38	-1	0.05
DAN	MDD	1	rs-fMRI	38	-1	0.05
Left Fronta	MDD	1	rs-fMRI	38	-1	0.05
Left Cereb	MDD	1	rs-fMRI	38	-1	0.05
Right Parie	MDD	1	rs-fMRI	38	-1	0.05
Right Fron	MDD	1	rs-fMRI	38	-1	0.05
ECN	DMN	1	rs-fMRI	38	-1	0.05
DAN	DMN	1	rs-fMRI	38	-1	0.05
Left Cereb	SN	1	rs-fMRI	38	-1	0.05
Right Cere	SN	1	rs-fMRI	38	-1	0.05
DMN	MDD	1	rs-fMRI	16	-1	0.05
SN	MDD	1	rs-fMRI	16	-1	0.05
Right Fron	MDD	1	rs-fMRI	16	1	0.05
Left Fronta	MDD	1	rs-fMRI	16	1	0.05
Right Insu	MDD	1	rs-fMRI	16	-1	0.05
Left Insula	MDD	1	rs-fMRI	16	-1	0.05
DMN	MDD	1	rs-fMRI	23	-1	0.05
ECN	MDD	1	rs-fMRI	23	1	0.05
SN	MDD	1	rs-fMRI	23	1	0.05
Left Temp	MDD	1	rs-fMRI	23	-1	0.05
Left Fronta	MDD	1	rs-fMRI	23	1	0.05
Left Pariet	MDD	1	rs-fMRI	23	1	0.05
Right Insul	MDD	1	rs-fMRI	23	1	0.05
Left Occip	MDD	1	rs-fMRI	23	-1	0.05
ECN	DMN	1	rs-fMRI	23	-1	0.05
SN	DMN	1	rs-fMRI	23	-1	0.05
DMN	MDD	1	rs-fMRI	41	-1	0.05
ON	MDD	1	rs-fMRI	41	1	0.05
Right Occi	MDD	1	rs-fMRI	41	1	0.05
Left Fronta	MDD	1	rs-fMRI	41	-1	0.05
Left Occip	MDD	1	rs-fMRI	41	-1	0.05
Left Temp	MDD	1	rs-fMRI	41	-1	0.05
DMN	MDD	1	rs-fMRI	11	-1	0.05
ECN	MDD	1	rs-fMRI	11	-1	0.05
ON	MDD	1	rs-fMRI	11	1	0.05
Right Occi	MDD	1	rs-fMRI	11	-1	0.05
Right Parie	MDD	1	rs-fMRI	11	-1	0.05
Left Temp	MDD	1	rs-fMRI	11	-1	0.05
Right Tem	MDD	1	rs-fMRI	11	-1	0.05
Right Fron	MDD	1	rs-fMRI	11	-1	0.05
Left Fronta	MDD	1	rs-fMRI	11	-1	0.05
ECN	DMN	1	rs-fMRI	11	-1	0.05
SN	DMN	1	rs-fMRI	11	-1	0.05
ON	DAN	1	rs-fMRI	11	-1	0.05
DMN	MDD	1	rs-fMRI	21	-1	0.05

SN	MDD	1	rs-fMRI	21	1	0.05
Left Fronta	MDD	1	rs-fMRI	21	1	0.05
Left Temp	MDD	1	rs-fMRI	21	1	0.05
Right Cere	MDD	1	rs-fMRI	21	-1	0.05
Right Pari	MDD	1	rs-fMRI	21	-1	0.05
Right Tem	MDD	1	rs-fMRI	21	-1	0.05
DAN	DMN	1	rs-fMRI	21	-1	0.05
ECN	DMN	1	rs-fMRI	21	1	0.05
Left Cereb	DMN	1	rs-fMRI	21	-1	0.05
DMN	MDD	1	rs-fMRI	42	-1	0.05
Right Tem	MDD	1	rs-fMRI	42	1	0.05
Left Temp	MDD	1	rs-fMRI	42	1	0.05
Right Pari	MDD	1	rs-fMRI	42	-1	0.05
Right Occi	MDD	1	rs-fMRI	42	-1	0.05
Right Fron	MDD	1	rs-fMRI	42	-1	0.05
Right Cere	MDD	1	rs-fMRI	42	-1	0.05
Left Pariet	MDD	1	rs-fMRI	42	-1	0.05
Left Occip	MDD	1	rs-fMRI	42	-1	0.05
Left Cereb	MDD	1	rs-fMRI	42	-1	0.05
Left Fronta	MDD	1	rs-fMRI	42	-1	0.05
DMN	MDD	1	rs-fMRI	37	-1	0.05
Right Occi	MDD	1	rs-fMRI	37	-1	0.05
Right Fron	MDD	1	rs-fMRI	37	1	0.05
Left Occip	MDD	1	rs-fMRI	37	-1	0.05
Left Fronta	MDD	1	rs-fMRI	37	1	0.05
DMN	MDD	1	rs-fMRI	32	-1	0.05
ON	MDD	1	rs-fMRI	32	1	0.05
Right Tem	MDD	1	rs-fMRI	32	1	0.05
Right Insu	MDD	1	rs-fMRI	32	1	0.05
Left Temp	MDD	1	rs-fMRI	32	1	0.05
Left Insula	MDD	1	rs-fMRI	32	1	0.05
Right Occi	MDD	1	rs-fMRI	32	-1	0.05
Right Fron	MDD	1	rs-fMRI	32	-1	0.05
Left Occip	MDD	1	rs-fMRI	32	-1	0.05
Left Fronta	MDD	1	rs-fMRI	32	-1	0.05
DMN	MDD	1	rs-fMRI	37	-1	0.05
Right Tem	MDD	1	rs-fMRI	37	-1	0.05
Left Temp	MDD	1	rs-fMRI	37	-1	0.05
DMN	MDD	1	rs-fMRI	21	-1	0.05
SN	MDD	1	rs-fMRI	21	-1	0.05
ECN	MDD	1	rs-fMRI	18	1	0.05
Left Fronta	MDD	1	rs-fMRI	18	1	0.05
Right Fron	MDD	1	rs-fMRI	18	1	0.05
DMN	MDD	1	rs-fMRI	7	-1	0.05
SN	MDD	1	rs-fMRI	7	-1	0.05
ECN	MDD	1	rs-fMRI	7	-1	0.05

Right Fron	MDD	1	rs-fMRI	7	1	0.05
Right Insul	MDD	1	rs-fMRI	7	-1	0.05
Left Fronta	MDD	1	rs-fMRI	7	-1	0.05
DMN	MDD	1	rs-fMRI	16	-1	0.05
ON	MDD	1	rs-fMRI	16	1	0.05
Right Tem	MDD	1	rs-fMRI	16	1	0.05
Left Temp	MDD	1	rs-fMRI	16	1	0.05
Right Fron	MDD	1	rs-fMRI	16	-1	0.05
Left Fronta	MDD	1	rs-fMRI	16	-1	0.05
Right Occi	MDD	1	rs-fMRI	16	-1	0.05
Left Occip	MDD	1	rs-fMRI	16	-1	0.05
DMN	MDD	1	rs-fMRI	24	-1	0.05
DAN	MDD	1	rs-fMRI	24	-1	0.05
Left Temp	MDD	1	rs-fMRI	24	-1	0.05
Left Pariet	MDD	1	rs-fMRI	24	-1	0.05
Right Tem	MDD	1	rs-fMRI	24	1	0.05
DMN	MDD	1	rs-fMRI	20	-1	0.05
SN	MDD	1	rs-fMRI	20	1	0.05
Left Insula	MDD	1	rs-fMRI	20	1	0.05
Right Tem	MDD	1	rs-fMRI	20	-1	0.05
Right Occi	MDD	1	rs-fMRI	20	-1	0.05
Right Fron	MDD	1	rs-fMRI	20	-1	0.05
Left Temp	MDD	1	rs-fMRI	20	-1	0.05
Left Occip	MDD	1	rs-fMRI	20	-1	0.05
Left Fronta	MDD	1	rs-fMRI	20	-1	0.05
DMN	MDD	1	rs-fMRI	22	-1	0.05
ON	MDD	1	rs-fMRI	22	1	0.05
Left Occip	MDD	1	rs-fMRI	22	1	0.05
Right Fron	MDD	1	rs-fMRI	22	-1	0.05
Left Temp	MDD	1	rs-fMRI	22	-1	0.05
Right Pari	MDD	1	rs-fMRI	22	-1	0.05
ECN	MDD	1	rs-fMRI	24	1	0.05
DMN	MDD	1	rs-fMRI	24	-1	0.05
Right Tem	MDD	1	rs-fMRI	24	-1	0.05
Left Fronta	MDD	1	rs-fMRI	24	1	0.05
Right Tem	MDD	1	rs-fMRI	24	-1	0.05
DMN	MDD	1	rs-fMRI	17	-1	0.05
ON	MDD	1	rs-fMRI	17	1	0.05
Right Pari	MDD	1	rs-fMRI	17	-1	0.05
Left Temp	MDD	1	rs-fMRI	17	-1	0.05
Right Tem	MDD	1	rs-fMRI	17	-1	0.05
Left Cereb	MDD	1	rs-fMRI	17	-1	0.05
Right Pari	MDD	1	rs-fMRI	17	-1	0.05
Right Tem	MDD	1	rs-fMRI	17	-1	0.05
DMN	MDD	1	rs-fMRI	15	-1	0.05
ECN	MDD	1	rs-fMRI	15	-1	0.05

ON	MDD	1	rs-fMRI	15	1	0.05
Left Temp	MDD	1	rs-fMRI	15	1	0.05
Left Cereb	MDD	1	rs-fMRI	15	1	0.05
Right Parie	MDD	1	rs-fMRI	15	1	0.05
Right Occi	MDD	1	rs-fMRI	15	-1	0.05
Left Fronta	MDD	1	rs-fMRI	15	-1	0.05
Right Fron	MDD	1	rs-fMRI	15	-1	0.05
DMN	MDD	1	rs-fMRI	49	-1	0.05
SN	MDD	1	rs-fMRI	49	1	0.05
ON	MDD	1	rs-fMRI	49	1	0.05
Left Fronta	MDD	1	rs-fMRI	49	-1	0.05
Right Fron	MDD	1	rs-fMRI	49	-1	0.05
Right Insu	MDD	1	rs-fMRI	49	1	0.05
Right Fron	MDD	1	rs-fMRI	49	-1	0.05
DMN	MDD	1	rs-fMRI	19	-1	0.05
ECN	MDD	1	rs-fMRI	19	-1	0.05
SN	MDD	1	rs-fMRI	19	1	0.05
Right Insu	MDD	1	rs-fMRI	19	1	0.05
Left Insula	MDD	1	rs-fMRI	19	1	0.05
Left Pariet	MDD	1	rs-fMRI	19	-1	0.05
Left Temp	MDD	1	rs-fMRI	19	-1	0.05
Left Fronta	MDD	1	rs-fMRI	19	-1	0.05
Right Fron	MDD	1	rs-fMRI	19	-1	0.05
Right Occi	MDD	1	rs-fMRI	19	-1	0.05
ECN	MDD	1	rs-fMRI	17	-1	0.05
DMN	MDD	1	rs-fMRI	17	-1	0.05
ON	MDD	1	rs-fMRI	17	1	0.05
Right Fron	MDD	1	rs-fMRI	17	-1	0.05
Left Fronta	MDD	1	rs-fMRI	17	-1	0.05
Left Temp	MDD	1	rs-fMRI	17	1	0.05
Right Tem	MDD	1	rs-fMRI	17	1	0.05
DMN	MDD	1	rs-fMRI	20	-1	0.05
SN	MDD	1	rs-fMRI	20	1	0.05
ECN	MDD	1	rs-fMRI	20	1	0.05
SN	DMN	1	rs-fMRI	11	-1	0.05
Left Fronta	DMN	1	rs-fMRI	11	-1	0.05
Left Pariet	DMN	1	rs-fMRI	11	-1	0.05
Right Parie	DMN	1	rs-fMRI	11	-1	0.05
Right Fron	DMN	1	rs-fMRI	11	-1	0.05
DMN	MDD	1	rs-fMRI	15	-1	0.05
SN	MDD	1	rs-fMRI	15	1	0.05
ON	MDD	1	rs-fMRI	15	1	0.05
DMN	MDD	1	rs-fMRI	44	-1	0.05
Right Fron	MDD	1	rs-fMRI	44	-1	0.05
Left Fronta	MDD	1	rs-fMRI	44	-1	0.05
DMN	MDD	1	rs-fMRI	15	-1	0.05

SN	MDD	1	rs-fMRI	15	-1	0.05
Left Pariet	MDD	1	rs-fMRI	15	1	0.05
Right Parie	MDD	1	rs-fMRI	15	1	0.05
Left Temp	MDD	1	rs-fMRI	15	-1	0.05
Left Fronta	MDD	1	rs-fMRI	15	-1	0.05
Left Cereb	MDD	1	rs-fMRI	15	-1	0.05
Right Insul	MDD	1	rs-fMRI	15	-1	0.05
DMN	MDD	1	rs-fMRI	15	-1	0.05
DMN	MDD	1	rs-fMRI	24	-1	0.05
Left Temp	MDD	1	rs-fMRI	24	-1	0.05
Left Fronta	MDD	1	rs-fMRI	24	1	0.05
DMN	MDD	1	rs-fMRI	20	-1	0.05
DAN	MDD	1	rs-fMRI	20	-1	0.05
SN	MDD	1	rs-fMRI	20	1	0.05
Right Insul	MDD	1	rs-fMRI	20	1	0.05
Left Insula	MDD	1	rs-fMRI	20	1	0.05
Left Pariet	MDD	1	rs-fMRI	20	1	0.05
Left Fronta	MDD	1	rs-fMRI	20	1	0.05
Right Parie	MDD	1	rs-fMRI	20	-1	0.05
Right Fron	MDD	1	rs-fMRI	20	-1	0.05
ON	MDD	1	rs-fMRI	17	1	0.05
Right Tem	MDD	1	rs-fMRI	17	1	0.05
Right Cere	MDD	1	rs-fMRI	17	1	0.05
Left Temp	MDD	1	rs-fMRI	17	1	0.05
Left Cereb	MDD	1	rs-fMRI	17	1	0.05
DMN	MDD	1	rs-fMRI	22	-1	0.05
SN	MDD	1	rs-fMRI	22	-1	0.05
ON	MDD	1	rs-fMRI	22	1	0.05
Left Temp	MDD	1	rs-fMRI	22	-1	0.05
Left Fronta	MDD	1	rs-fMRI	22	-1	0.05
Right Tem	MDD	1	rs-fMRI	22	-1	0.05
Right Fron	MDD	1	rs-fMRI	22	-1	0.05
Right Insul	MDD	1	rs-fMRI	22	-1	0.05
Right Occi	MDD	1	rs-fMRI	22	-1	0.05
DMN	MDD	1	rs-fMRI	14	-1	0.05
ECN	MDD	1	rs-fMRI	14	1	0.05
Left Cereb	MDD	1	rs-fMRI	14	1	0.05
Left Fronta	MDD	1	rs-fMRI	14	1	0.05
Left Occip	MDD	1	rs-fMRI	14	-1	0.05
Right Occi	MDD	1	rs-fMRI	14	-1	0.05
Left Temp	MDD	1	rs-fMRI	14	-1	0.05
Right Fron	MDD	1	rs-fMRI	14	-1	0.05
DMN	MDD	1	rs-fMRI	44	-1	0.05
Right Fron	MDD	1	rs-fMRI	44	-1	0.05
Left Fronta	MDD	1	rs-fMRI	44	1	0.05
Right Parie	MDD	1	rs-fMRI	44	-1	0.05
