# Supplementary material for: Probabilistic Entity-Relationship Diagram: A correlation between functional connectivity and spontaneous brain activity during resting state in major depressive disorder
Source: PLoS One. 2017 Jun 8;12(6):e0178386. doi: 10.1371/journal.pone.0178386 (PMC5464553; doi:10.1371/journal.pone.0178386)
Supplement: S2 Appendix — (PDF) [file pone.0178386.s002.pdf]

major fac	related fa	cross/lon	imaging	No of No	corrlatio	p value
DMN	MDD	1	rs-fMRI	38	-1	0.05
DAN	MDD	1	rs-fMRI	38	-1	0.05
Left Fron	MDD	1	rs-fMRI	38	-1	0.05
Left Cere	MDD	1	rs-fMRI	38	-1	0.05
Right Par	MDD	1	rs-fMRI	38	-1	0.05
Right Fro	MDD	1	rs-fMRI	38	-1	0.05
ECN	DMN	1	rs-fMRI	38	-1	0.05
DAN	DMN	1	rs-fMRI	38	-1	0.05
Left Cere	SN	1	rs-fMRI	38	-1	0.05
Right Ce	SN	1	rs-fMRI	38	-1	0.05
DMN	MDD	1	rs-fMRI	16	-1	0.05
SN	MDD	1	rs-fMRI	16	-1	0.05
Right Fro	MDD	1	rs-fMRI	16	1	0.05
Left Fron	MDD	1	rs-fMRI	16	1	0.05
Right Ins	MDD	1	rs-fMRI	16	-1	0.05
Left Insu	MDD	1	rs-fMRI	16	-1	0.05
DMN	MDD	1	rs-fMRI	23	-1	0.05
ECN	MDD	1	rs-fMRI	23	1	0.05
SN	MDD	1	rs-fMRI	23	1	0.05
Left Tem	MDD	1	rs-fMRI	23	-1	0.05
Left Fron	MDD	1	rs-fMRI	23	1	0.05
Left Pari	MDD	1	rs-fMRI	23	1	0.05
Right Ins	MDD	1	rs-fMRI	23	1	0.05
Left Occi	MDD	1	rs-fMRI	23	-1	0.05
ECN	DMN	1	rs-fMRI	23	-1	0.05
SN	DMN	1	rs-fMRI	23	-1	0.05
DMN	MDD	1	rs-fMRI	41	-1	0.05
ON	MDD	1	rs-fMRI	41	1	0.05
Right Oc	MDD	1	rs-fMRI	41	1	0.05
Left Fron	MDD	1	rs-fMRI	41	-1	0.05
Left Occi	MDD	1	rs-fMRI	41	-1	0.05
Left Tem	MDD	1	rs-fMRI	41	-1	0.05
DMN	MDD	1	rs-fMRI	11	-1	0.05
ECN	MDD	1	rs-fMRI	11	-1	0.05
ON	MDD	1	rs-fMRI	11	1	0.05
Right Oc	MDD	1	rs-fMRI	11	-1	0.05
Right Par	MDD	1	rs-fMRI	11	-1	0.05
Left Tem	MDD	1	rs-fMRI	11	-1	0.05

Right Te	MDD	1	rs-fMRI	11	-1	0.05
Right Fro	MDD	1	rs-fMRI	11	-1	0.05
Left Fron	MDD	1	rs-fMRI	11	-1	0.05
ECN	DMN	1	rs-fMRI	11	-1	0.05
SN	DMN	1	rs-fMRI	11	-1	0.05
ON	DAN	1	rs-fMRI	11	-1	0.05
DMN	MDD	1	rs-fMRI	21	-1	0.05
SN	MDD	1	rs-fMRI	21	1	0.05
Left Fron	MDD	1	rs-fMRI	21	1	0.05
Left Tem	MDD	1	rs-fMRI	21	1	0.05
Right Ce	MDD	1	rs-fMRI	21	-1	0.05
Right Pa	MDD	1	rs-fMRI	21	-1	0.05
Right Te	MDD	1	rs-fMRI	21	-1	0.05
DAN	DMN	1	rs-fMRI	21	-1	0.05
ECN	DMN	1	rs-fMRI	21	1	0.05
Left Cere	DMN	1	rs-fMRI	21	-1	0.05
DMN	MDD	1	rs-fMRI	42	-1	0.05
Right Te	MDD	1	rs-fMRI	42	1	0.05
Left Tem	MDD	1	rs-fMRI	42	1	0.05
Right Pa	MDD	1	rs-fMRI	42	-1	0.05
Right Oc	MDD	1	rs-fMRI	42	-1	0.05
Right Fro	MDD	1	rs-fMRI	42	-1	0.05
Right Ce	MDD	1	rs-fMRI	42	-1	0.05
Left Pari	MDD	1	rs-fMRI	42	-1	0.05
Left Occi	MDD	1	rs-fMRI	42	-1	0.05
Left Cere	MDD	1	rs-fMRI	42	-1	0.05
Left Fron	MDD	1	rs-fMRI	42	-1	0.05
DMN	MDD	1	rs-fMRI	37	-1	0.05
Right Oc	MDD	1	rs-fMRI	37	-1	0.05
Right Fro	MDD	1	rs-fMRI	37	1	0.05
Left Occi	MDD	1	rs-fMRI	37	-1	0.05
Left Fron	MDD	1	rs-fMRI	37	1	0.05
DMN	MDD	1	rs-fMRI	32	-1	0.05
ON	MDD	1	rs-fMRI	32	1	0.05
Right Te	MDD	1	rs-fMRI	32	1	0.05
Right Ins	MDD	1	rs-fMRI	32	1	0.05
Left Tem	MDD	1	rs-fMRI	32	1	0.05
Left Insu	MDD	1	rs-fMRI	32	1	0.05
Right Oc	MDD	1	rs-fMRI	32	-1	0.05

Right Fro	MDD	1	rs-fMRI	32	-1	0.05
Left Occi	MDD	1	rs-fMRI	32	-1	0.05
Left Fron	MDD	1	rs-fMRI	32	-1	0.05
DMN	MDD	1	rs-fMRI	37	-1	0.05
Right Te	MDD	1	rs-fMRI	37	-1	0.05
Left Tem	MDD	1	rs-fMRI	37	-1	0.05
DMN	MDD	1	rs-fMRI	21	-1	0.05
SN	MDD	1	rs-fMRI	21	-1	0.05
ECN	MDD	1	rs-fMRI	18	1	0.05
Left Fron	MDD	1	rs-fMRI	18	1	0.05
Right Fro	MDD	1	rs-fMRI	18	1	0.05
DMN	MDD	1	rs-fMRI	7	-1	0.05
SN	MDD	1	rs-fMRI	7	-1	0.05
ECN	MDD	1	rs-fMRI	7	-1	0.05
Right Fro	MDD	1	rs-fMRI	7	1	0.05
Right Ins	MDD	1	rs-fMRI	7	-1	0.05
Left Fron	MDD	1	rs-fMRI	7	-1	0.05
DMN	MDD	1	rs-fMRI	16	-1	0.05
ON	MDD	1	rs-fMRI	16	1	0.05
Right Te	MDD	1	rs-fMRI	16	1	0.05
Left Tem	MDD	1	rs-fMRI	16	1	0.05
Right Fro	MDD	1	rs-fMRI	16	-1	0.05
Left Fron	MDD	1	rs-fMRI	16	-1	0.05
Right Oc	MDD	1	rs-fMRI	16	-1	0.05
Left Occi	MDD	1	rs-fMRI	16	-1	0.05
DMN	MDD	1	rs-fMRI	24	-1	0.05
DAN	MDD	1	rs-fMRI	24	-1	0.05
Left Tem	MDD	1	rs-fMRI	24	-1	0.05
Left Pari	MDD	1	rs-fMRI	24	-1	0.05
Right Te	MDD	1	rs-fMRI	24	1	0.05
DMN	MDD	1	rs-fMRI	20	-1	0.05
SN	MDD	1	rs-fMRI	20	1	0.05
Left Insu	MDD	1	rs-fMRI	20	1	0.05
Right Te	MDD	1	rs-fMRI	20	-1	0.05
Right Oc	MDD	1	rs-fMRI	20	-1	0.05
Right Fro	MDD	1	rs-fMRI	20	-1	0.05
Left Tem	MDD	1	rs-fMRI	20	-1	0.05
Left Occi	MDD	1	rs-fMRI	20	-1	0.05
Left Fron	MDD	1	rs-fMRI	20	-1	0.05
