# Supplementary material for: Probabilistic Entity-Relationship Diagram: A correlation between functional connectivity and spontaneous brain activity during resting state in major depressive disorder
Source: PLoS One. 2017 Jun 8;12(6):e0178386. doi: 10.1371/journal.pone.0178386 (PMC5464553; doi:10.1371/journal.pone.0178386)
Supplement: S3 Appendix — (PDF) [file pone.0178386.s003.pdf]

major factor	related facto	cross/longitu	imaging mo	No of Norm	corrlation va	p value
DMN	MDD	1	rs-fMRI	22	-1	0.05
ON	MDD	1	rs-fMRI	22	1	0.05
Left Occipita	MDD	1	rs-fMRI	22	1	0.05
Right Fronta	MDD	1	rs-fMRI	22	-1	0.05
Left Tempora	MDD	1	rs-fMRI	22	-1	0.05
Right Parieta	MDD	1	rs-fMRI	22	-1	0.05
ECN	MDD	1	rs-fMRI	24	1	0.05
DMN	MDD	1	rs-fMRI	24	-1	0.05
Right Tempo	MDD	1	rs-fMRI	24	-1	0.05
Left Frontal I	MDD	1	rs-fMRI	24	1	0.05
Right Tempo	MDD	1	rs-fMRI	24	-1	0.05
DMN	MDD	1	rs-fMRI	17	-1	0.05
ON	MDD	1	rs-fMRI	17	1	0.05
Right Parieta	MDD	1	rs-fMRI	17	-1	0.05
Left Tempora	MDD	1	rs-fMRI	17	-1	0.05
Right Tempo	MDD	1	rs-fMRI	17	-1	0.05
Left Cerebel	MDD	1	rs-fMRI	17	-1	0.05
Right Parieta	MDD	1	rs-fMRI	17	-1	0.05
Right Tempo	MDD	1	rs-fMRI	17	-1	0.05
DMN	MDD	1	rs-fMRI	15	-1	0.05
ECN	MDD	1	rs-fMRI	15	-1	0.05
ON	MDD	1	rs-fMRI	15	1	0.05
Left Tempora	MDD	1	rs-fMRI	15	1	0.05
Left Cerebel	MDD	1	rs-fMRI	15	1	0.05
Right Parieta	MDD	1	rs-fMRI	15	1	0.05
Right Occipit	MDD	1	rs-fMRI	15	-1	0.05
Left Frontal I	MDD	1	rs-fMRI	15	-1	0.05
Right Fronta	MDD	1	rs-fMRI	15	-1	0.05
DMN	MDD	1	rs-fMRI	49	-1	0.05
SN	MDD	1	rs-fMRI	49	1	0.05
ON	MDD	1	rs-fMRI	49	1	0.05
Left Frontal I	MDD	1	rs-fMRI	49	-1	0.05
Right Fronta	MDD	1	rs-fMRI	49	-1	0.05
Right Insular	MDD	1	rs-fMRI	49	1	0.05
Right Fronta	MDD	1	rs-fMRI	49	-1	0.05
DMN	MDD	1	rs-fMRI	19	-1	0.05
ECN	MDD	1	rs-fMRI	19	-1	0.05
SN	MDD	1	rs-fMRI	19	1	0.05
Right Insular	MDD	1	rs-fMRI	19	1	0.05
Left Insular	MDD	1	rs-fMRI	19	1	0.05
Left Parietal	MDD	1	rs-fMRI	19	-1	0.05
Left Tempora	MDD	1	rs-fMRI	19	-1	0.05
Left Frontal I	MDD	1	rs-fMRI	19	-1	0.05
Right Fronta	MDD	1	rs-fMRI	19	-1	0.05
Right Occipit	MDD	1	rs-fMRI	19	-1	0.05
ECN	MDD	1	rs-fMRI	17	-1	0.05

DMN	MDD	1	rs-fMRI	17	-1	0.05
ON	MDD	1	rs-fMRI	17	1	0.05
Right Fronta	MDD	1	rs-fMRI	17	-1	0.05
Left Frontal I	MDD	1	rs-fMRI	17	-1	0.05
Left Tempora	MDD	1	rs-fMRI	17	1	0.05
Right Tempo	MDD	1	rs-fMRI	17	1	0.05
DMN	MDD	1	rs-fMRI	20	-1	0.05
SN	MDD	1	rs-fMRI	20	1	0.05
ECN	MDD	1	rs-fMRI	20	1	0.05
SN	DMN	1	rs-fMRI	11	-1	0.05
Left Frontal I	DMN	1	rs-fMRI	11	-1	0.05
Left Parietal	DMN	1	rs-fMRI	11	-1	0.05
Right Parieta	DMN	1	rs-fMRI	11	-1	0.05
Right Fronta	DMN	1	rs-fMRI	11	-1	0.05
DMN	MDD	1	rs-fMRI	15	-1	0.05
SN	MDD	1	rs-fMRI	15	1	0.05
ON	MDD	1	rs-fMRI	15	1	0.05
DMN	MDD	1	rs-fMRI	44	-1	0.05
Right Fronta	MDD	1	rs-fMRI	44	-1	0.05
Left Frontal I	MDD	1	rs-fMRI	44	-1	0.05
DMN	MDD	1	rs-fMRI	15	-1	0.05
SN	MDD	1	rs-fMRI	15	-1	0.05
Left Parietal	MDD	1	rs-fMRI	15	1	0.05
Right Parieta	MDD	1	rs-fMRI	15	1	0.05
Left Tempora	MDD	1	rs-fMRI	15	-1	0.05
Left Frontal I	MDD	1	rs-fMRI	15	-1	0.05
Left Cerebel	MDD	1	rs-fMRI	15	-1	0.05
Right Insular	MDD	1	rs-fMRI	15	-1	0.05
DMN	MDD	1	rs-fMRI	15	-1	0.05
DMN	MDD	1	rs-fMRI	24	-1	0.05
Left Tempora	MDD	1	rs-fMRI	24	-1	0.05
Left Frontal I	MDD	1	rs-fMRI	24	1	0.05
DMN	MDD	1	rs-fMRI	20	-1	0.05
DAN	MDD	1	rs-fMRI	20	-1	0.05
SN	MDD	1	rs-fMRI	20	1	0.05
Right Insular	MDD	1	rs-fMRI	20	1	0.05
Left Insular	MDD	1	rs-fMRI	20	1	0.05
Left Parietal	MDD	1	rs-fMRI	20	1	0.05
Left Frontal I	MDD	1	rs-fMRI	20	1	0.05
Right Parieta	MDD	1	rs-fMRI	20	-1	0.05
Right Fronta	MDD	1	rs-fMRI	20	-1	0.05
ON	MDD	1	rs-fMRI	17	1	0.05
Right Tempo	MDD	1	rs-fMRI	17	1	0.05
Right Cerebe	MDD	1	rs-fMRI	17	1	0.05
Left Tempora	MDD	1	rs-fMRI	17	1	0.05
Left Cerebel	MDD	1	rs-fMRI	17	1	0.05
DMN	MDD	1	rs-fMRI	22	-1	0.05

SN	MDD	1	rs-fMRI	22	-1	0.05
ON	MDD	1	rs-fMRI	22	1	0.05
Left Tempor	MDD	1	rs-fMRI	22	-1	0.05
Left Frontal I	MDD	1	rs-fMRI	22	-1	0.05
Right Tempo	MDD	1	rs-fMRI	22	-1	0.05
Right Fronta	MDD	1	rs-fMRI	22	-1	0.05
Right Insular	MDD	1	rs-fMRI	22	-1	0.05
Right Occipit	MDD	1	rs-fMRI	22	-1	0.05
DMN	MDD	1	rs-fMRI	14	-1	0.05
ECN	MDD	1	rs-fMRI	14	1	0.05
Left Cerebel	MDD	1	rs-fMRI	14	1	0.05
Left Frontal I	MDD	1	rs-fMRI	14	1	0.05
Left Occipita	MDD	1	rs-fMRI	14	-1	0.05
Right Occipit	MDD	1	rs-fMRI	14	-1	0.05
Left Tempor	MDD	1	rs-fMRI	14	-1	0.05
Right Fronta	MDD	1	rs-fMRI	14	-1	0.05
DMN	MDD	1	rs-fMRI	44	-1	0.05
Right Fronta	MDD	1	rs-fMRI	44	-1	0.05
Left Frontal I	MDD	1	rs-fMRI	44	1	0.05
Right Parieta	MDD	1	rs-fMRI	44	-1	0.05
